# ^177^Lu-PSMA-617 Therapy in Mice, with or without the Antioxidant α_1_-Microglobulin (A1M), Including Kidney Damage Assessment Using ^99m^Tc-MAG3 Imaging

**DOI:** 10.3390/biom11020263

**Published:** 2021-02-10

**Authors:** Amanda Kristiansson, Anders Örbom, Jonas Ahlstedt, Helena Karlsson, Wahed Zedan, Magnus Gram, Bo Åkerström, Sven-Erik Strand, Mohamed Altai, Joanna Strand, Oskar Vilhelmsson Timmermand

**Affiliations:** 1 Section for Infection Medicine, Department of Clinical Sciences, Lund University, 221 84 Lund, Sweden; bo.akerstrom@med.lu.se; 2 Division of Hematology and Transfusion Medicine, Department of Laboratory Medicine, Lund University, 221 84 Lund, Sweden; 3 Division of Oncology, Department of Clinical Sciences Lund, Lund University, 221 00 Lund, Sweden; anders.orbom@med.lu.se (A.Ö.); wahed.zedan@med.lu.se (W.Z.); sven-erik.strand@med.lu.se (S.-E.S.); mohamed.altai@med.lu.se (M.A.); joanna.strand@med.lu.se (J.S.); oskar.vilhelmsson_timmermand@med.lu.se (O.V.T.); 4 Lund University Bioimaging Center, Lund University, 221 84 Lund, Sweden; jonas.ahlstedt@med.lu.se; 5 Pediatrics, Department of Clinical Sciences Lund, Lund University, 221 84 Lund, Sweden; helena.karlsson@med.lu.se (H.K.); magnus.gram@med.lu.se (M.G.); 6 Division of Medical Radiation Physics, Department of Clinical Sciences Lund, Lund University, 221 00 Lund, Sweden

**Keywords:** radioligand therapy, α_1_-microglobulin, [^99m^Tc]Tc-MAG3 imaging, [^177^Lu]Lu-PSMA-617, prostate cancer, mouse model, kidney damage, dosimetry

## Abstract

Anti-prostate specific membrane antigen (PSMA) radioligand therapy is promising but not curative in castration resistant prostate cancer. One way to broaden the therapeutic index could be to administer higher doses in combination with radioprotectors, since administered radioactivity is kept low today in order to avoid side-effects from a high absorbed dose to healthy tissue. Here, we investigated the human radical scavenger α_1_-microglobulin (A1M) together with 177-Lutetium (^177^Lu) labeled PSMA-617 in preclinical models with respect to therapeutic efficacy and kidney toxicity. Nude mice with subcutaneous LNCaP xenografts were injected with 50 or 100 MBq of [^177^Lu]Lu-PSMA-617, with or without injections of recombinant A1M (rA1M) (at T = 0 and T = 24 h). Kidney absorbed dose was calculated to 7.36 Gy at 4 days post a 100 MBq injection. Activity distribution was imaged with Single-Photon Emission Computed Tomography (SPECT) at 24 h. Tumor volumes were measured continuously, and kidneys and blood were collected at termination (3–4 days and 3–4 weeks after injections). In a parallel set of experiments, mice were given [^177^Lu]Lu-PSMA-617 and rA1M as above and dynamic technetium-99m mercaptoacetyltriglycine ([^99m^Tc]Tc-MAG3) SPECT imaging was performed prior to injection, and 3- and 6-months post injection. Blood and urine were continuously sampled. At termination (6 months) the kidneys were resected. Biomarkers of kidney function, expression of stress genes and kidney histopathology were analyzed. [^177^Lu]Lu-PSMA-617 uptake, in tumors and kidneys, as well as treatment efficacy did not differ between rA1M and vehicle groups. In mice given rA1M, [^99m^Tc]Tc-MAG3 imaging revealed a significantly higher slope of initial uptake at three months compared to mice co-injected with [^177^Lu]Lu-PSMA-617 and vehicle. Little or no change compared to control was seen in urine albumin, serum/plasma urea levels, RT-qPCR analysis of stress response genes and in the kidney histopathological evaluation. In conclusion, [^99m^Tc]Tc-MAG3 imaging presented itself as a sensitive tool to detect changes in kidney function revealing that administration of rA1M has a potentially positive effect on kidney perfusion and tubular function when combined with [^177^Lu]Lu-PSMA-617 therapy. Furthermore, we could show that rA1M did not affect anti-PSMA radioligand therapy efficacy.

## 1. Introduction

Prostate cancer (PCa) is among the most common malignancies in men. Initially all PCa tumors are androgen responsive and thus androgen deprivation therapy (ADT) is the first line therapy of choice with a high response rate in metastatic disease. However, treatment with ADT progresses the disease to a castration resistant state called castration resistant prostate cancer (CRPC). In the STAMPEDE trial, the median failure free survival for patients with metastatic androgen sensitive PCa treated with ADT alone was 11.2 months [1].

One relatively new treatment modality is targeting of prostate cancer with prostate specific membrane antigen (PSMA) specific radiolabeled ligands. PSMA is a transmembrane protein not only expressed in PCa but also in other malignancies such as triple negative breast, thyroid and renal clear cell carcinoma [2,3,4]. PSMA radioligand therapy has been successful in prolonging the life and temporarily reducing the tumor burden in terminally ill prostate cancer patients with limited observed toxicity, although it is not curative in metastatic CRPC [5,6,7]. All published clinical studies to date have been conducted as salvage therapy or followed patients with an overall survival of approximately one year; hence, the relatively good toxicity data reported do not necessarily take into account eventual late effects of the treatment. Therefore, uptake in kidney and salivary glands must be seen as a potential obstacle for the realization of broader use of PSMA specific radioligands.

Peptide-receptor radionuclide therapy (PRRT) using radionuclide labeled somatostatin analogues, e.g., 177-Lutetium (^177^Lu) labeled (DOTA)0-Tyr3octreotate (DOTATATE), is frequently used today to treat metastasized neuroendocrine tumors [8]. Renal toxicity has been observed following PRRT, due to filtration and reabsorption of radioligands in the kidneys, with an elevated risk in patients suffering from poor renal function, e.g., from chemotherapy, hypertension or diabetes [9,10,11,12].

The classical absorbed dose limit for the whole kidney is 23 Gy, derived from effects seen in external radiotherapy [13]. It has long been understood that higher absorbed doses can be tolerated during radionuclide therapy and a Biologically Effective Dose (BED) limit of 28 Gy for patients at risk and 40 Gy to the kidneys in risk-free patients has been suggested by Bodei et al. [10]. In a study on patients treated with ^177^Lu-DOTATATE by Bergsma et al. little toxicity was seen in patients with a kidney absorbed dose exceeding 28 Gy concluding that this is a rather conservative value [14]. So far, in patients receiving ^177^Lu-labeled PSMA-617 ([^177^Lu]Lu-PSMA-617) therapy, kidneys have received as much as 30 Gy without nephrotoxicity above grade 2 being observed for up to 12 weeks after the last treatment cycle. Escalating tumor absorbed doses to efficiently combat the disease, might surpass what is today considered the kidney absorbed dose limit. A strategy to do so could involve the use of kidney radioprotectors, such as α_1_-microglobulin (A1M).

A1M is a 26 kDa human protein with antioxidant, heme-binding, radical-scavenging and reductase properties [15,16,17]. It is mainly synthesized in the liver and has a plasma concentration of 20–50 mg/L with a half-life of 2–3 min and clearance via the kidneys [18,19,20,21]. A1M has been shown to protect against oxidative damage in cells, tissues and organs, including kidneys [22,23,24,25,26,27,28]. Interestingly, an in vitro study showed reduction in cell death and oxidation markers by A1M in α-particle irradiated cells and bystander cells, emphasizing the role of oxidative stress in radiation damage [29]. Moreover, in a recent study exogenously injected A1M, shown to be mainly localized to the kidney cortex [30], protected the kidneys against radiotoxicity during ^177^Lu-DOTATATE treatment in a mouse model. A follow-up study later established that the tumor treatment itself was not affected by A1M [30,31,32,33].

Taken together, these effects make A1M a highly interesting radioprotection candidate for use in combination with radiolabeled PSMA ligands. A1M may prevent the long-term adverse effects of the treatment and/or allow for a broader therapeutic window, i.e., higher injected activities or increase in number of fractions given.

In this study, we investigated the effects of [^177^Lu]Lu-PSMA-617, 50 and 100 MBq/mouse, alone or co-administered with recombinant A1M (rA1M) in two preclinical mouse models. Kidney function was evaluated using Technetium-99m (^99m^Tc) mercaptoacetyltriglycine (MAG3) imaging as well as standard kidney toxicity markers and methodologies.

## 2. Materials and Methods

### 2.1. Radiolabeling

Radiolabeling was conducted as previously described by Kratochwil et al. [34]. 1 GBq of non-carrier added Lutetium-177 (ITG GmbH, Garching, Germany), 30–40 MBq/µL, was mixed with 100 µL sterile 0.4 M sodium acetate solution, pH 5.5, and 1.25 µL 20% *w*/*w* ascorbic acid solution; thereafter, 2 µL of a 10 mM solution of PSMA-617 (ABX, Radeberg, Germany) was added. The solution was heated to and maintained at 95 °C on a shaker for 15 min. Samples, 1 µL, were taken for instant Thin Layer Chromatography (iTLC) at 0 and 15 min. The iTLC strips were placed in 0.2 M sodium citrate solution (mobile phase), which was allowed to migrate up the strip. In this system the free, unbound, ^177^Lu migrates with the solvent front of the mobile phase. The iTLC strips were then analyzed on a phosphor imager system (Cyclone Plus Phosphor Imager, PerkinElmer, Inc., Waltham, MA, USA). The reaction was terminated by removing the sample from the shaker and cooling it down to room temperature. A sterile 0.9% sodium chloride solution was added, and the radiotracer diluted (1:3 or 1:7), a sample for iTLC taken, and pH tested, before injections in mice.

### 2.2. Cell Culturing

The prostate cancer-cell line LNCaP-FGC was purchased from the American Type Culture Collection (ATCC^®^, Manassas, VA, USA) and cultured as monolayer using RPMI-1640 medium with L-glutamine (Biowest, Nuaillé, France) supplemented with 10% Fetal Bovine Serum (Fisher Scientific, Loughborough, UK) and 1% penicillin-streptomycin (Fisher Scientific). The cells were grown in incubators with 37 °C, 5% CO_2_ and in a humidified atmosphere. The cells were screened for mycoplasma (mycoplasma check, Eurofins GATC Biotech, Konstanz, Germany) regularly and before inoculation. The isogenic human prostate carcinoma cell line PC3-pip (PSMA positive) was obtained from Professor Anna Orlova, Uppsala University, and cultured as described above for LNCaP-FGC.

### 2.3. In Vivo Studies

In vivo studies were conducted in both tumor bearing and non-tumor bearing mice (See Table 1). All experiments were conducted in accordance with the directions given by the regional ethical committee for animal trials. BALB/cAnNRj mice (Janvier Labs, Le Genest-Saint-Isle, France) were injected with 100 MBq of [^177^Lu]Lu-PSMA-617 (n = 15) or 50 MBq of [^177^Lu]Lu-PSMA-617 (n = 15) together with vehicle or 100 MBq (n = 15) or 50 MBq (n = 15) of [^177^Lu]Lu-PSMA-617 together with 5 mg/kg of rA1M (Guard Therapeutics International AB, Lund, Sweden) followed by an additional injection of rA1M (5 mg/kg) after 24 h. The volume of radiotracer injected per mice was 50 µL and the volume of vehicle or rA1M was 50 µL at each injection. One group (n = 15) was injected with rA1M and vehicle. These mice were sacrificed and dissected with urine and kidney collected and terminal blood drawn after 6 months.

BALB/cAnNRj-Foxn1*^nu/nu^* mice were inoculated subcutaneously on their right flank with 200 µL of a 1:1 RPMI-1640 and Matrigel (BD Biosciences, San Jose, CA, USA) solution containing 4–6 million LNCaP cells. The tumors were allowed to establish for about 3 weeks reaching a size of 5–8 mm in diameter. These mice were then injected with 100 MBq of [^177^Lu]Lu-PSMA-617 together with vehicle (n = 14) or 100 MBq together with 5 mg/kg of rA1M followed by an additional injection of rA1M (5 mg/kg) after 24 h (n = 14). One group of mice received rA1M together with vehicle (n = 7). Mice were euthanized at 3–4 days or 3–4 weeks after injections. Blood, urine, tumor and kidney was sampled. Before and during treatment with [^177^Lu]Lu-PSMA-617 the xenografts in the different groups were monitored by caliper measurements twice every week.

### 2.4. [99^m^Tc]Tc-MAG3 and [^177^Lu]Lu-PSMA-617 Imaging

A Single-Photon Emission Computed Tomography with Computed Tomography (SPECT/CT) (NanoSPECT/CT Plus, Mediso; Budapest, Hungary) equipped with the NSP-106 multipinhole mouse collimator was used for all imaging and SPECT data was reconstructed using HiSPECT software (SciVis, Goettingen, Germany) and the “Standard” preset. Imaging of kidney Technetium-99m (^99m^Tc) mercaptoacetyltriglycine (MAG3) accumulation was conducted in 21 of the BALB/cAnNRj mice before injections of [^177^Lu]Lu-PSMA-617 and rA1M. After 3 and 6 months, [^99m^Tc]Tc-MAG3 was injected and SPECT/CT imaging was conducted. Mice were anesthetized with 2–3% isoflurane (Baxter, Deerfield, IL, USA) in a O_2_ and N_2_O mix. First a full body CT was conducted, and detectors positioned over the kidneys with the help of the CT image. A semi-stationary ^99m^Tc SPECT protocol (140 keV ± 20%) was used with a frame rate of 5 s and a total of 78 recorded frames. After the first five frames 200 µL of [^99m^Tc]Tc-MAG3 (31 ± 7 MBq) were injected through a catheter with a syringe pump (PhD 1000, Harvard Apparatus, Holliston, MA, USA).

Imaging tumor uptake of [^177^Lu]Lu-PSMA-617 in eight tumor bearing mice from each group (100 MBq of [^177^Lu]Lu-PSMA-617 together with vehicle or 100 MBq together with rA1M, see above) was done with SPECT/CT 24 h after injection. Energy windows of 20% was centered over the 56-, 113-, and 208-keV energy peaks of ^177^Lu.

Based on the SPECT signal, regions of interest (ROI) where drawn using the VivoQuant 3.0 software (inviCRO; Boston, MA, USA). Activity data (MBq/mm^3^) were extracted and then converted to percent injected activity per gram of tissue (% IA/g) by assuming a tissue density of 1.0 g/cm^3^. For [^99m^Tc]Tc-MAG3 imaging all frames where first merged so that a ROI could be drawn separately over the renal pelvis and another over the renal cortex and medulla as well as a background ROI below the kidney. Thereafter, the image with the merged frames was closed and all frames reopened, and ROI data extracted. When analyzing the data T = 0 was determined as the fifth frame, that is the first 5 s before injection.

### 2.5. WBC, RBC and Platelet Counts

Four days after co-injection of 100 MBq of [^177^Lu]Lu-PSMA-617 with rA1M or vehicle in LNCaP tumor bearing mice, 20 µL of blood were collected from the tail vein. Total white blood cell counts (WBC), red blood cell counts (RBC) and platelets (PLT) were then meadured in an Exigo Veterinary (Exigo Vet) Hematology Analyzer (Boule Medical, Stockholm, Sweden).

### 2.6. Functional Urine and Serum/Plasma Markers

Blood was collected from vena saphena or as terminal blood by heart puncture from mice. Blood was then transferred to serum or plasma tubes. Serum tubes were allowed to coagulate for 20–60 min and were then spun down at 3000 rpm for 10 min after which serum was collected. Plasma tubes were spun down almost immediately, using the same speed and time as the serum tubes; thereafter, plasma was collected. The samples were then transferred to dry ice and later stored at −80 °C. Albumin concentrations in urine were analyzed using a mouse albumin simple-step ELISA kit (Abcam, Cambridge, UK) according to the instructions provided by the manufacturer. Urine and serum creatinine concentrations were analyzed using QuantiChrom Creatinine Assay Kit according to instructions from manufacturer (BioAssay Systems, Hayward, CA, USA). Serum/plasma urea was analyzed with the QuantiChrom Urea Assay Kit (BioAssay Systems). A1M concentrations in serum/plasma and urine were measured with radioimmunoassay as previously described [35], using recombinant mouse A1M and rabbit anti-recombinant mouse A1M prepared in our laboratory as described [36]. Radiolabeling of A1M with ^125^I was done using the chloramine T method [37].

### 2.7. RT-qPCR in Kidney Tissue

Total RNA was isolated from frozen renal tissue stored in RNAlater (Thermo Fischer, Waltham, MA, USA) using the RNeasy Mini Kit (QIAGEN, Valencia, CA, USA) including DNAse treatment (QIAGEN). Whole kidney samples were used for the extraction. Extracted RNA was reverse-transcribed into cDNA using the iScript cDNA synthesis kit (Bio-Rad, Hercules, CA, USA). mRNA expression of Ngal (Lcn2) (Eurofins), A1M (Ambp) (QIAGEN), Hmox1 (QIAGEN) and Hsp70 (Hspa1b) (QIAGEN) were analyzed in renal tissue and normalized against the control gene β-actin (Eurofins) using the iTaq Universal SYBR Green Supermix (Bio-Rad). DNA amplification was performed using thermocycling conditions as recommended by the manufacturers, starting with an initial polymerase inactivation step at 95 °C for 3 min followed by 40 cycles 95 °C for 5 s and 55 °C for 30 s for Eurofins primers or for QIAGEN primers 95 °C for 10 min followed by 40 cycles 95 °C for 15 s and 60 °C for 60 s (CFX Connect Real-Time PCR Detection System, Bio-Rad). Data is presented as fold change values (2^−ΔΔCT^ method).

### 2.8. Statistical Analysis of Functional Urine and Serum/Plasma Markers and RT-qPCR

Data are presented as mean ± SD if not stated otherwise. Statistical significance was calculated with a one-way ANOVA test corrected for multiple comparisons (Sidak) or with an unpaired *t*-test. Statistical analysis was performed with GraphPad Prism (GraphPad Prism 8.3.0 for MacOS; GraphPad Software; GraphPad, San Diego, CA, USA). Values of *p* <  0.05 were considered significant.

### 2.9. Histopathology

Resected kidneys were put in 4% paraformaldehyde overnight and then dehydrated and embedded in paraffin. Paraffin sections, 4 µm thick, of the kidneys including the renal medulla, cortex and pelvis were rehydrated, stained with hematoxylin and eosin and then dehydrated and mounted. The lesions in the kidneys were then scored by a pathologist as previously described [32].

### 2.10. Dosimetry

Dynamic SPECT imaging was performed with one frame per minute from the point of injection to 120 min post injection of BALB/c male mice injected with either 200 MBq (n = 4) or 100 MBq (n = 4) of [^177^Lu]Lu-PSMA-617 (n = 8) to obtain a rough estimate of the absorbed dose to kidneys in the mice. Peptide mass was 17 nmol for 200 MBq and 2 nmol for 100 MBq. Four of the animals received co-injection with 5 mg/kg rA1M but no difference in uptake was observed; consequently, all data was pooled in analysis. A few later time points were also imaged by SPECT, 8 h (n = 1), 10 h (n = 1) and 13 h (n = 1). To account for possible inaccuracies in SPECT quantification at these levels of activity, biodistribution measurements were performed where the same type of animals were injected with 50 MBq of [^177^Lu]Lu-PSMA-617, again with approximately 1 nmol of peptide mass, and sacrificed at 10 min (n = 2), 43 min (n = 1), 46 min (n = 1) and 24 h (n = 2) post injection and measured the activities in the kidneys. Each SPECT image was normalized to these measurements and mean values at each time point were used to create a model uptake in the kidneys over time. Due to practical difficulties of obtaining this data, data from Umbricht et al. [38] was used to estimate data for 48 h and 96 h post injection. Measurements for kidney dosimetry were made using PSMA-617 acquired from a new vendor (MedChemExpress, Monmouth Junction, NJ, USA) as the previous one had ceased to offer it. The specificity of the PSMA-617 from the new vendor was confirmed by measuring in vitro uptake in PSMA-positive PC3-pip cells which was 90% without blocking with excess PSMA-617 and 5% with blocking.

## 3. Results

### 3.1. [^177^Lu]Lu-PSMA-617 and [^99m^Tc]Tc-MAG3 Imaging

**Figure 1:**
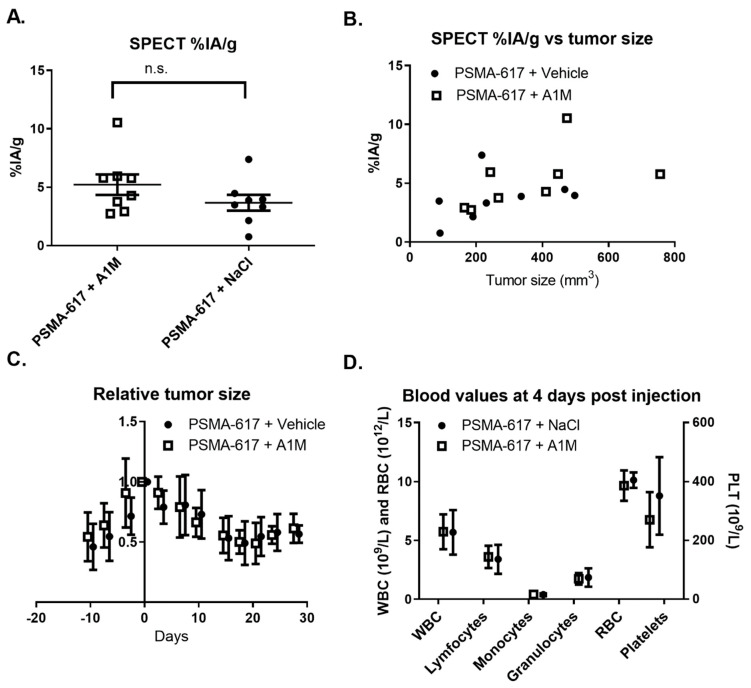
Therapeutic efficacy following administration of 100 MBq of [^177^Lu]Lu-PSMA-617 with vehicle or with recombinant α_1_-microglobulin (rA1M) (5 mg/kg). (**A**) [^177^Lu]Lu-PSMA-617 tumor uptake 24 h post injection, quantified from single photon emission computed tomography (SPECT) images, in mice given rA1M or vehicle (Data presented as mean ± SEM). The uptake was 5.2 ± 2.3 percent injected activity per gram (% IA/g) (n = 8) for mice treated with [^177^Lu]Lu-PSMA-617 and rA1M compared to 3.6 ± 1.8% IA/g (n = 8) for mice treated with [^177^Lu]Lu-PSMA-617 and vehicle, the difference was not significant (n.s.). (**B**) Tumor uptake as a function of tumor size. (**C**) Treatment response of the LNCaP xenografts over time, presented as the relative tumor size compared to tumor volume at start (mean ± SD). (**D**) White blood cells (WBC), red blood cells (RBC) and platelet (PLT) counts for mice treated with [^177^Lu]Lu-PSMA-617 given rA1M or vehicle (mean ± SD).

Mice with LNCaP tumors injected with 100 MBq of [^177^Lu]-PSMA-617, with or without (5 mg/kg) rA1M, imaged 24 h after injection using SPECT/CT showed no significant differences in [^177^Lu]Lu-PSMA-617 tumor uptake (Figure 1A) and displayed a similar relationship between the uptake and tumor size (Figure 1B). Furthermore, no differences in the relative tumor size, as measured by caliper, were observed during the following 28 days post injection (Figure 1C). Finally, no significant differences in blood cell counts between the two groups were observed (Figure 1D).

**Figure 2:**
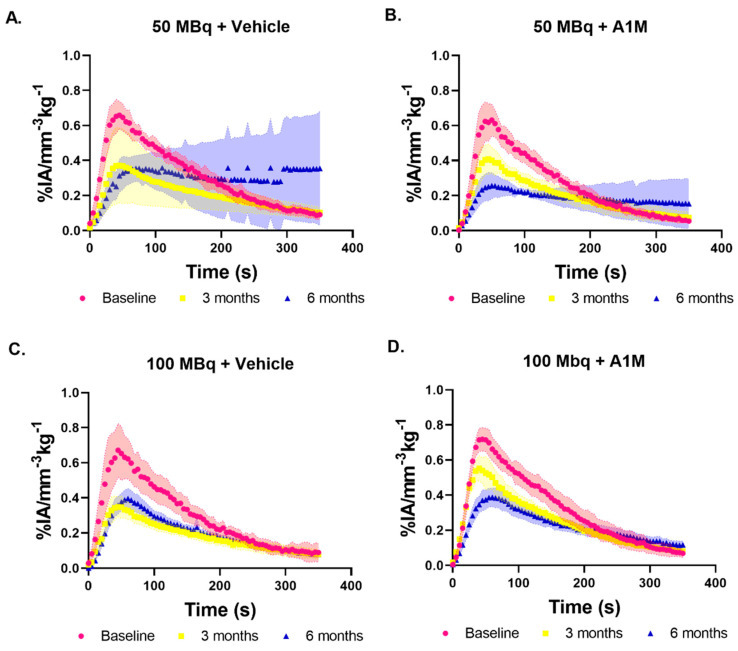
Average kidney time activity curves (TACs), with standard deviation, following [^99m^Tc]Tc-MAG3 imaging at baseline (red), three (yellow) and six (blue) months post treatment with (**A**) 50 MBq of [^177^Lu]Lu-PSMA-617 (Baseline, n = 6; three months, n = 5; six months, n = 4), (**B**) 50 MBq of [^177^Lu]Lu-PSMA-617 with rA1M (Baseline, n = 6; three months, n = 4; six months, n = 4), (**C**) 100 MBq of [^177^Lu]Lu-PSMA-617 (Baseline, n = 6; three months, n = 7; six months, n = 6), and (**D**) 100 MBq of [^177^Lu]Lu-PSMA-617 with rA1M (Baseline, n = 6; three months, n = 4; six months, n = 4).

**Figure 3:**
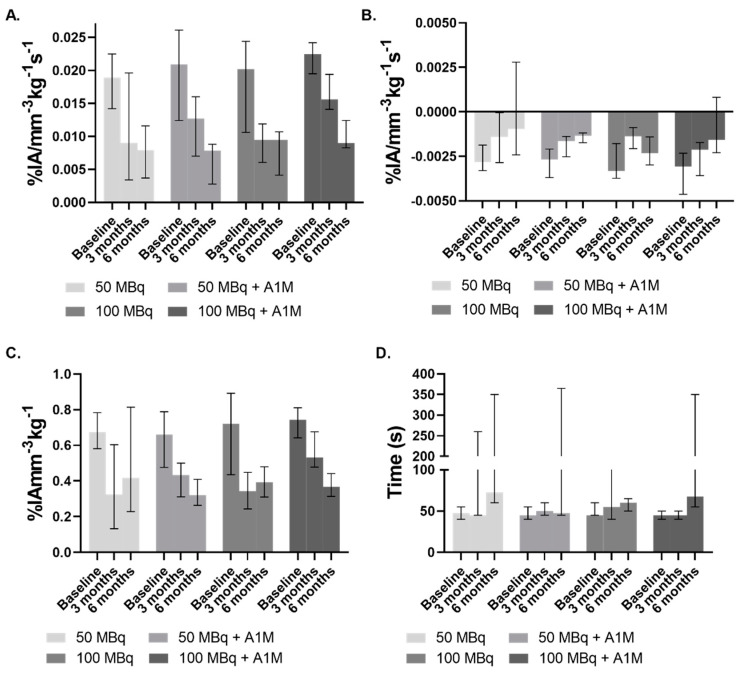
[^99m^Tc]Tc-MAG3 kidney TACs median and range. (**A**) The slope of initial uptake (SIU) i.e., the inclination the first 5–35 s. (**B**) Excretion in all groups over time. (**C**) TAC peak uptake values. (**D**) Time to peak.

[^99m^Tc]Tc-MAG3 imaging in mice without tumor was conducted prior to injection (Baseline) and at 3- and 6-months post injection of [^177^Lu]-PSMA-617, with vehicle or with rA1M (5 mg/kg). The resulting average renograms, or time activity curves (TACs) are presented in Figure 2. The TACs were analyzed with regards to the time to peak, peak value, inclination during the first 5–35 s, slope of initial uptake (SIU) [39], and the 65–125 s inclination (excretion), and these values are presented with median and range in Figure 3. At baseline there is little difference between the respective activity groups with overall similar values for all the analyzed parameters, though the mice that received 100 MBq with rA1M have a smaller range (Figure 3A). The two groups of mice given rA1M have a higher SIU median at three months than those not given rA1M and their range display almost no overlap with their six months data (Figure 3A). This in contrast to the groups without rA1M, which have a similar median and overlapping range at three and six months. Comparison show that mice given 100 MBq of [^177^Lu]Lu-PSMA-617 with rA1M had a mean SIU (16 × 10^−3^ ± 2 × 10^−3^% IA mm^−3^ kg^−1^ s^−1^) that was significantly higher (*p* = 0.005) than the corresponding group without rA1M (8 × 10^−3^ ± 3 × 10^−3^% IA mm^−3^ kg^−1^ s^−1^) at three months. Interestingly, with regards to SIU, there is no difference between the groups at six months. SIU is an important measurement as it depends on both the perfusion, uptake and retention as well as excretion. The median slope during the isolated excretion phase (65–125 s), reflecting renal or urethral blockage, deteriorate over time in all groups. Mice exposed to 50 MBq of [^177^Lu]Lu-PSMA-617 seem to be the least affected with regards to both median and range (Figure 3B), whereas, surprisingly, there is an indication towards higher values in the 100 MBq group at 6 months. This can also be seen in the TACs (Figure 2). At three months, mice given 100 MBq with rA1M have significantly higher peak values (*p* = 0.01) compared to those given 100 MBq [^177^Lu]Lu-PSMA-617 and vehicle (Figure 3C), and compared to both baseline and the 100 MBq group, retained time to peak (Figure 3D). This is not as pronounced in the groups given 50 MBq with vehicle or with rA1M; however, the 50 MBq with vehicle group displays larger variation, as compared to the 100 MBq vehicle group, with regards to both peak value and time to peak (Figure 3A,C).

At six months, the SIU and peak values had deteriorated for all groups and were in a similar range. To evaluate this further, we imaged mice, administered with only vehicle, with [^99m^Tc]Tc-MAG3 SPECT/CT (n = 6), which showed that the SIU (median, 9.2 × 10^−3^% IA mm^−3^ kg^−1^ s^−1^; range, 1.3 × 10^−2^–8.0 × 10^−3^% IA mm^−3^ kg^−1^ s^−1^) was similar to the other groups (Figure 3). This was also true for peak values (median, 0.36% IA mm^−3^ kg^−1^; range, 0.48–0.32% IA mm^−3^ kg^−1^). However, with regards to excretion, this group performed better with values (median, −2.4 × 10^−3^% IA mm^−3^ kg^−1^ s^−1^; range, −1.48 × 10^−3^–−2.93 × 10^−3^% IA mm^−3^ kg^−1^ s^−1^) similar to those at baseline (Figure 3B). This was also true with regards to time to peak (median, 55 s; range, 50–65 s) where all [^177^Lu]Lu-PSMA-617 treated groups showed an elevated time to peak or large variability (Figure 3D). Furthermore, almost all treated groups, and most pronounced in mice given 50 MBq only, contain mice that show a lack of MAG3 excretion resulting in TACs similar to that of mice imaged using [^99m^Tc]Tc-MAG3 with unilateral ureteral obstruction in a study by Tantawy et al. [39] and this is probably the main reason for the large variation seen in time to peak and also influencing the average kidney TACs (Figure 2).

**Figure 4:**
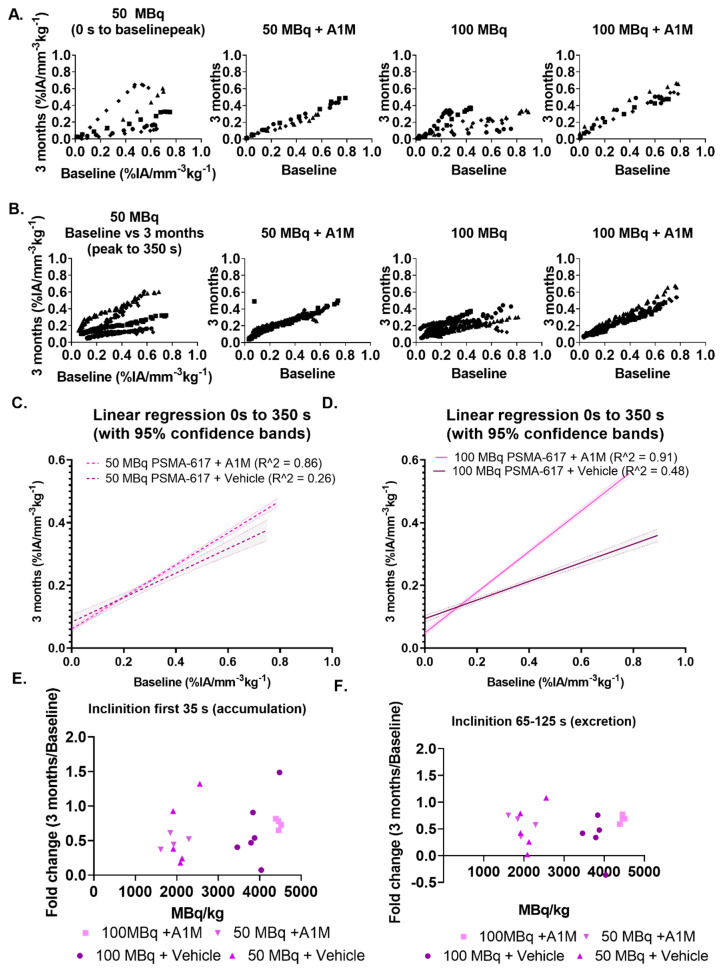
Three months [^99m^Tc]Tc-MAG3 activity data normalized to each corresponding individual baseline [^99m^Tc]Tc-MAG3 activity data. (**A**) The normalized initial uptake phase for rA1M and vehicle treated groups. (**B**) The normalized excretion phase. (**C**) Linear regression of the normalized SIU values and normalized excretion phase for 50 MBq [^177^Lu]Lu-PSMA-617. (**D**) Linear regression of the normalized SIU values and normalized excretion phase for 100 MBq [^177^Lu]Lu-PSMA-617. (**E**) Normalized slope of the accumulation phase when plotting these values vs. activity concentration MBq/kg. (**F**) Normalized slope of the excretion phase.

Examining individual changes in relation to their baseline measurements was done by plotting the baseline values (% IA mm^−3^ kg^−1^) obtained from [^99m^Tc]Tc-MAG3 prior to injection of [^177^Lu]Lu-PSMA-617 against the corresponding, in seconds post injection of [^99m^Tc]Tc-MAG3, values obtained three months post injection, with the baseline peak as cut-off for accumulation and excretion. In both individual data and in the total cohort data, mice treated with rA1M (5 mg/kg) displayed a linear pattern (Figure 4A,B), whereas the groups without rA1M display a large variation (Figure 4A,B). Linear regression on the combined data from each group given rA1M results in a R^2^ of 0.91 (100 MBq) and 0.86 (50 MBq) as compared to 0.48 (100 MBq) and 0.26 (50 MBq) in the groups without rA1M (Figure 4C,D). The slope of the line for respective groups are 0.39 ± 0.03 (50 MBq), 0.51 ± 0.01 (50 MBq + rA1M), 0.29 ± 0.02 (100 MBq) and 0.65 ± 0.01 (100 MBq + rA1M). Similarly, the larger variability in mice not given rA1M, can be seen by plotting the fold change (three months vs. baseline) of the SIU and the inclination at 65–125 s (excretion) vs. the injected activity normalized to bodyweight, a common way to predict toxicity (Figure 4E,F).

### 3.2. Biochemical Kidney Damage Markers, Gene Expression and Histopathology

Albumin levels in urine, a marker for renal glomerular damage, were measured after eight days, one and a half, respectively, six months post-injection in non-tumor bearing animals and after three- and 28-days post-injection in mice with LNCaP tumors. There were no clear trends in the data, with only a significant increase of albumin levels in animals receiving 100 MBq with vehicle after eight days (Supplementary Figure S1A–E). A1M in urine, a marker of kidney tubular damage, was measured at 6 months post-injection and showed no difference between the groups receiving 50 or 100 MBq with or without rA1M. A1M was also measured in serum at six months post-injection in non-tumor bearing mice and at 28 days post-injection in tumor bearing mice, without detecting any differences (Supplementary Figure S1F–H). Similar to A1M, serum creatinine levels at 6 months displayed no differences between the groups (Supplementary Figure S2).

Urea is a waste product eliminated by the kidneys, and an increase in serum or plasma is strongly associated with reduction in glomerular filtration rate. In this study, levels were measured after eight days post-injection in addition to three- and six-months post-injection in non-tumor bearing animals and after three- and 28-days post-injection in mice with tumors (Supplementary Figure S1J–N). A small increase in serum urea was seen after 6 months post-injection in the group receiving 50 MBq but at the earlier timepoint no changes could be detected. In the tumor bearing animals, animals not receiving tumor treatment with [^177^Lu]Lu-PSMA-617 showed an increase in urea levels at three days post [^177^Lu]Lu-PSMA-617 administration (Supplementary Figure S1M).

Renal tissue gene expression was analyzed in the mice with LNCaP tumors that had been injected with [^177^Lu]Lu-PSMA-617, with vehicle or with rA1M. Animals sacrificed after three or 28 days displayed no significant differences in mRNA levels of the stress related genes Hmox-1 and Hspa1a between groups that had received rA1M or not (Supplementary Figure S2A,B,E,F). Furthermore, no difference in mRNA levels between groups was observed when evaluating the kidney damage marker Ngal or the gene encoding A1M, Ambp (Supplementary Figure S2C,D,G,H).

Histopathological evaluation of renal tissue at six months showed no significant differences between the groups, rA1M vs. vehicle.

### 3.3. Dosimetry

**Figure 5:**
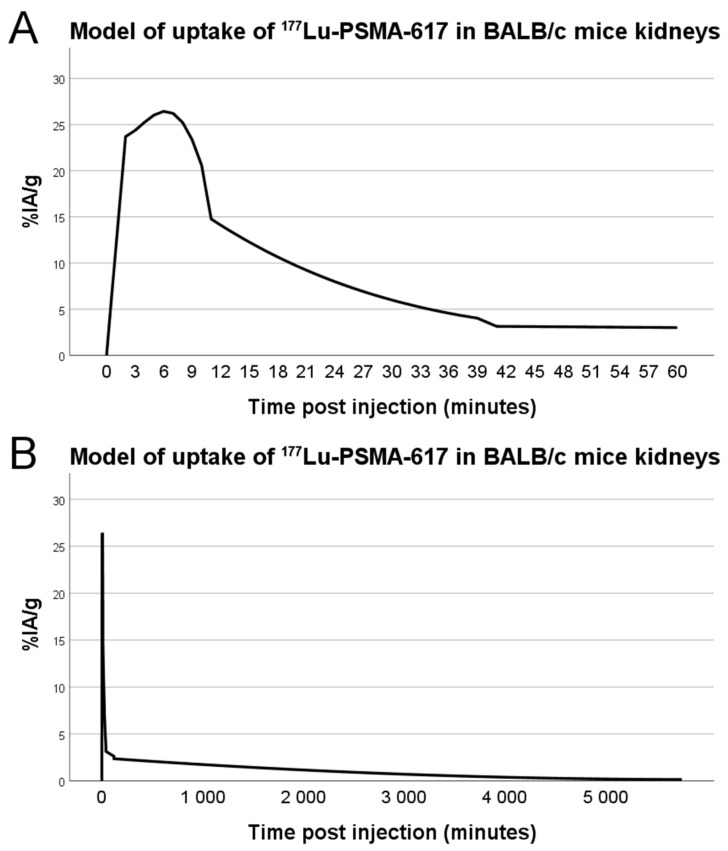
Model of uptake of [^177^Lu]Lu-PSMA-617 in the kidneys of BALB/c male mice based on in vivo dynamic SPECT imaging (n = 8), ex vivo biodistribution measurements (n = 6) and literature data for 1-, 2- and 4-days post injection. Initial uptake (**A**) and long-time retention (**B**).

From the dynamic SPECT measurements, biodistribution data, as well as employing biodistribution for late time-points from Umbricht et al. [38], and linear interpolation between data points, we created a model for the kidney uptake over time, displayed in Figure 5. Correcting for radionuclide decay, we used the data together with Kidney to Kidney S values for ^177^Lu in mice from Larsson et al. [40]. This resulted in an absorbed dose to kidneys of 0.07 Gy per injected MBq at 4 days post injection, consequently 3.68 Gy for 50 MBq and 7.36 Gy for 100 MBq.

## 4. Discussion

The combination of radiotherapy with radioprotectors to protect healthy organs raises the question of the influence of the radioprotectors on therapeutic efficacy. We investigated the potential influence on uptake from co-injections of rA1M and [^177^Lu]Lu-PSMA-617 (Figure 1A,B) as well as the effect on tumor growth and bone marrow toxicity (Figure 1C,D). This showed that rA1M does not interfere with the uptake nor therapeutic effect of [^177^Lu]Lu-PSMA-617 in our preclinical model of prostate cancer as no significant differences could be seen between mice given vehicle or rA1M together with [^177^Lu]Lu-PSMA-617. This is in line with previously published results by Andersson et al. [33] who combined [^177^Lu]-octreotate with rA1M in a model of neuroendocrine tumors.

Initial estimations of absorbed dose to kidneys were made based on the 2015 paper by Benešová et al. [41]. From this paper we could use measured values for kidney uptake of [^177^Lu]Lu-PSMA-617 at 1 h (137.2% IA/g) and 24 h post injection, but for the first-hour uptake we assumed that the uptake had the same shape, but not amplitude, as the ^68^Ga-PSMA-617 which they had measured with dynamic PET imaging. Based on this data and assumptions, we estimated that the mouse kidneys would receive 0.55 Gy per injected MBq. However, later publications have recorded much lower kidney uptake of [^177^Lu]Lu-PSMA-617, with the highest uptake being around 30% IA/g at 15 min post injection [38,42,43]. Our own measurements were even slightly below these numbers at early time points and above them at 24 h, which could be partly due to differences in experimental setup, data analysis of SPECT images or handling of excised tissues. We used higher amounts of radioactivity, and for some animals a slightly higher peptide mass, when doing SPECT imaging for dosimetry in order to capture enough image statistics in the dynamic studies, although this can of course also introduce errors. We used literature data for uptake at 48 h and 96 h post injection for our dosimetry model which could introduce errors since those data [38] were based on studies using PC-3 tumor-bearing female BALB/c mice, not non-tumor male BALB/c, like in our study. However, the injected peptide mass of about 1 nmol in those studies should be close to ours. The quantitative measurements of activity in SPECT images were not used, only the relative amounts of uptake between different time points. This was due to surprisingly low values observed in the images, possibly due to dead-time errors, and since no known source was included in the image for calibration, we opted to go for using only the shape of the curve and calibrating to ex vivo biodistribution. Further careful characterization of the absorbed dose to kidneys in mouse models of PSMA-617 therapy is necessary to provide a detailed picture, although a rough estimate like in our case can at least differentiate between a dose close to either a full treatment or that of a single therapy fraction.

In humans, a renal BED limit of 28 Gy for patients at risk and 40 Gy to the risk-free patients have been suggested [10]. We have not exposed the mouse kidneys to close to those clinical limits in this study, reaching at most 7.36 Gy for the 100 MBq groups. However, as clinical absorbed doses are usually split into fractions, i.e., several smaller absorbed doses, the absorbed dose in this study is in a range similar to one fraction given in the clinic [34,42,43,44,45].

These absorbed doses most likely explain why we could not see any significant differences in serum, plasma and urine functional markers as well as kidney gene expression analysis (Supplementary Figures S1 and S2). This is not unexpected as the absorbed dose was low and potential damage to the kidneys is complex and involve healing.

[^99m^Tc]Tc-MAG3 has previously been reported to be more sensitive in detecting tubular damage in mice than both biochemical and histological measurements in ischemia/reperfusion injury models [46]. In the current study, we wanted to investigate the use of [^99m^Tc]Tc-MAG3 imaging to detect changes in a medium- to long-term (3–6 months) study following therapy with [^177^Lu]Lu-PSMA-617. PSMA-617 has been suggested to be reabsorbed in the proximal tubule [47], which could lead to high absorbed doses locally in this compartment. The calculated absorbed doses reported here are mean absorbed doses for the whole kidney volume, thus any heterogeneity of activity distribution would likely result in much higher absorbed doses locally. [^99m^Tc]Tc-MAG3 functional imaging should be preferable when studying tubular function as tubular excretion is the main route of MAG3 elimination, whereas, e.g., diethylenetriamine pentaacetic acid (DTPA) is mainly excreted via glomeruli filtration. It is in fact the proximal tubule cells that are responsible for excreting MAG3 [48]; thus, the effects on MAG3 accumulation and excretion seen by us in this study could be linked to the damage resulting from [^177^Lu]Lu-PSMA-617 absorbed dose to the kidneys. It should be noted that the activities from administration of [^99m^Tc]Tc-MAG3 can in itself give a dose dependent radiation-effect to the kidneys and it cannot be excluded that it can contribute to kidney damage.

The large variations, in some cases already at the baseline measurements, raises the question of how to analyze these data with the individual differences in kidney function taken into account, something that may be further complicated by the normal physiological changes in kidney function over time. However, the SIU is reliant on both uptake and excretion and is therefore a good measure of overall kidney function. As mice given rA1M performs better with regards to SIU at three months we decided to investigate this further.

The data on relative kidney function, measured as SIU (Figure 3A), indicate that rA1M improves the renal function following exposure to 50 MBq [^177^Lu]Lu-PSMA-617. However, it does not allow any conclusions whether the kidney function changes seen are due to a more severe damage as this could not be confirmed with standard methods of kidney damage (Supplementary Figure S1). Analysis of the SIU data, a combined measure of the uptake and excretion, i.e., a measure of perfusion and the relative kidney function [46], indicate that mice given rA1M have an improved perfusion and function of the kidneys at three months. As there is individual variability at the baseline measurement, we analyzed the relative change in the SIU and the excretion by plotting the baseline values (% IA mm^−3^ kg^−1^ s^−1^) vs. those at three months based on the baseline peak. Surprisingly, mice given rA1M had a more uniform relationship between their baseline and three months values (Figure 4A). Moreover, mice given rA1M had a higher slope in both activity groups (Figure 4C,D), which is in line with the conclusion that these animals suffered less change in kidney function as compared to animals without rA1M (vehicle group).

rA1M seems to benefit kidney perfusion and possibly tubular function following co-injection with [^177^Lu]Lu-PSMA-617. Our data show that mice treated with rA1M during [^177^Lu]Lu-PSMA-617 have a higher SIU at three months than mice not treated with rA1M. These changes are detected with [^99m^Tc]Tc-MAG3 imaging but were not observed with more traditional tools to measure kidney function. This suggests that [^99m^Tc]Tc-MAG3 may be a more sensitive indicator of aberrations in the affected kidney parameters, organ perfusion and tubular reabsorption, when using low absorbed doses. Furthermore, we observed that rA1M improved the renal function as evaluated by [^99m^Tc]Tc-MAG3 imaging. However, considering that traditional biochemical and histopathological evaluation parameters were not significantly affected by [^177^Lu]Lu-PSMA-617 exposure, more preclinical work is needed to conclude that rA1M is actually preventing the negative effects of [^177^Lu]Lu-PSMA-617. The absorbed dose given in this study comes close to the absorbed dose clinically given to the kidney at each fraction of PSMA therapy (~6–8 Gy) [34,42,43,44,45]. An animal model that is treated in several fractions with activities at or over 100 MBq [^177^Lu]Lu-PSMA-617 per fraction could provide absorbed doses to the kidney closer to the clinical limit.

## 5. Conclusions

In conclusion, mice given rA1M showed a better kidney perfusion at three months, which could be an effect of its function as a radioprotector of the kidneys. Furthermore, no negative effects on tumor therapy efficacy were seen in mice given rA1M, which suggests that rA1M does not interfere with the therapeutic potential of [^177^Lu]Lu-PSMA-617. The absorbed dose to the mouse kidney close to a single fractionated absorbed dose was too low to compare to the full fractionated clinical situation, so further studies with full fractionation are needed to confirm the protective effect of rA1M.

## 6. Patents

B.Å. and M.G. are a co-founders and share-holders of Guard Therapeutics International AB, which holds the patent rights for medical use of A1M.

## Figures and Tables

**Table 1:** Preclinical study outline.

Strain	Xenografted	100 MBq + rA1M	100MBq + Vehicle	50 MBq + rA1M	50 Mbq + Vehicle
BALB/cAnNRj	No	n = 15	n = 15	n = 15	n = 15
BALB/cAnNRj-Foxn1^nu/nu^	LNCaP cells	n = 14	n = 14	-	-
	**[^99m^Tc]Tc-MAG3 SPECT**	**[^177^Lu]Lu-PSMA-617 SPECT**	**Blood Cell Counts**	**RT-qPCR**	**Functional Urine and Serum/Plasma Markers**
BALB/cAnNRj	Yes	Yes (kidney dosimetry)	No	Yes	Yes
BALB/cAnNRj-Foxn1^*nu*/*nu*^	No	Yes (tumor uptake)	Yes	Yes	Yes

## Data Availability

Data will be available on request.

## Supplementary Materials

The following are available online at https://www.mdpi.com/2218-273X/11/2/263/s1, Figure S1: Biochemical kidney damage markers, Figure S2: mRNA levels in kidney tissue of stress and damage related genes in tumor bearing mice.
