# Supplementary material for: 177Lu-PSMA-617 Therapy in Mice, with or without the Antioxidant α1-Microglobulin (A1M), Including Kidney Damage Assessment Using 99mTc-MAG3 Imaging
Source: Biomolecules. 2021 Feb 10;11(2):263. doi: 10.3390/biom11020263 (PMC7916794; doi:10.3390/biom11020263)
Supplement: Supplementary file 1 [file biomolecules-11-00263-s001.pdf]

Supplementary Materials

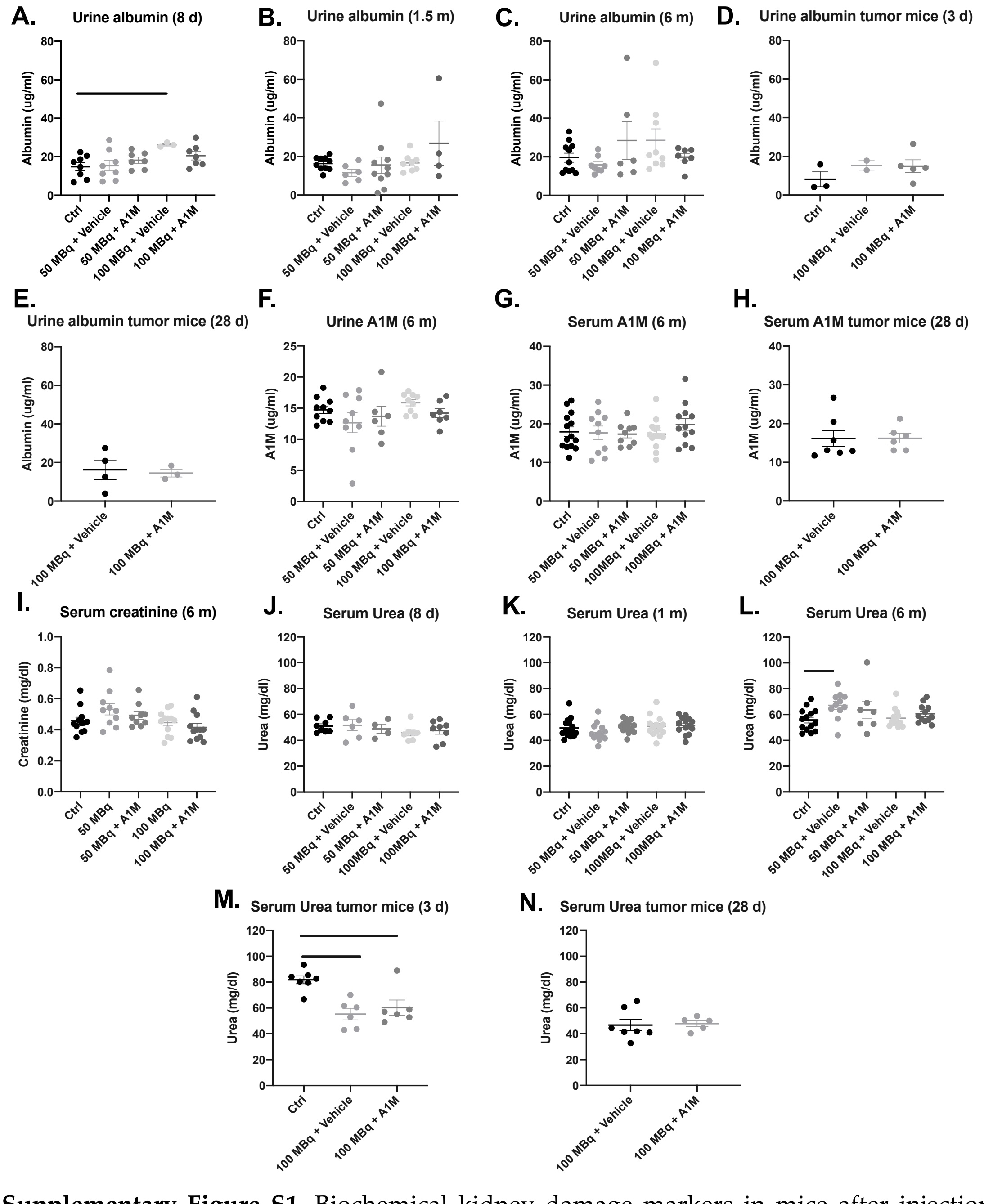

Supplementary Figure S1. Biochemical kidney damage markers in mice after injection of ^{177}Lu-PSMA-617 with or without co-administration of A1M. Albumin in urine sampled at (**A**) 8 days, (**B**) 1.5 months, (**C**) 6 months in mice and (**D**) 3 days and (**E**) 28 days in tumor bearing mice. Endogenous A1M levels after 6 months in (**F**) urine and (**G**) serum and (**H**) serum A1M in tumor bearing mice after 28 days. Creatinine levels in serum after (**I**) 6 months. Urea in serum sampled at (**A**) 8 days, (**B**) 1 months, (**C**) 6 months in non-tumor bearing mice and (**D**) 3 days and (**E**) 28 days in tumor bearing mice. Data is presented as mean ± SEM (One-way ANOVA with Sidak multiple comparison test for three groups or more and unpaired *t*-test for two groups).

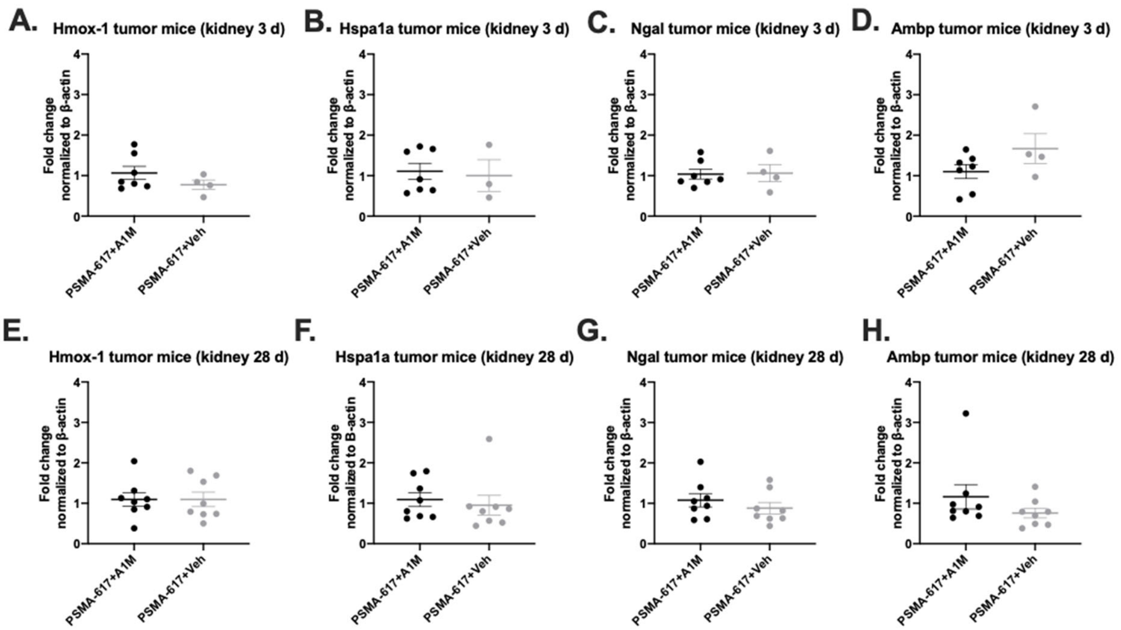

Supplementary Figure S2. mRNA levels in kidney tissue of stress and damage related genes in tumor bearing mice after injection of 100 MBq of ^{177}Lu-PSMA-617 with or without co-administration of A1M (5mg/kg). Expression of (**A**) Hmox-1, (**B**) Hsp1a, (**C**) Ngal and (**D**) Ambp after three days. Expression of (**E**) Hmox-1, (**F**) Hsp1a, (**G**) Ngal and (**H**) Ambp after 28 days. Data is presented as fold change (mean ± SEM) vs. ^{177}Lu-PSMA-617 + A1M group.
